# Low-level laser irradiation effect on endothelial cells under conditions of hyperglycemia

**DOI:** 10.1007/s10103-016-1880-4

**Published:** 2016-02-09

**Authors:** Krzysztof Góralczyk, Justyna Szymańska, Katarzyna Szot, Jacek Fisz, Danuta Rość

**Affiliations:** Department of Pathophysiology, Faculty of Pharmacy, Nicolaus Copernicus University in Toruń, Collegium Medicum in Bydgoszcz, Skłodowskiej-Curie Street No 9, Bydgoszcz, Poland; Department of Laserotherapy and Physiotherapy, Faculty of Health Sciences, Nicolaus Copernicus University in Toruń, Collegium Medicum in Bydgoszcz, Bydgoszcz, Poland

**Keywords:** Low-level laser therapy, TNF-alpha, IL-6, Endothelial cells

## Abstract

Diabetes mellitus is considered to be a very serious lifestyle disease leading to cardiovascular complications and impaired wound healing observed in the diabetic foot syndrome. Chronic hyperglycemia is the source of the endothelial activation. The inflammatory process in diabetes is associated with the secretion of inflammatory cytokines by endothelial cells, e.g., tumor necrosis factor-alpha (TNF-α) and interleukin 6 (IL-6). The method of phototherapy using laser beam of low power (LLLT—low-level laser therapy) effectively supports the conventional treatment of diabetic vascular complications such as diabetic foot syndrome. The aim of our study was to evaluate the effect of low-power laser irradiation at two wavelengths (635 and 830 nm) on the secretion of inflammatory factors (TNF-α and IL-6) by the endothelial cell culture—HUVEC line (human umbilical vein endothelial cell)—under conditions of hyperglycemia. It is considered that adverse effects of hyperglycemia on vascular endothelial cells may be corrected by the action of LLLT, especially with the wavelength of 830 nm. It leads to the reduction of TNF-α concentration in the supernatant and enhancement of cell proliferation. Endothelial cells play an important role in the pathogenesis of diabetes; however, a small number of studies evaluate an impact of LLLT on these cells under conditions of hyperglycemia. Further work on this subject is warranted.

## Introduction

In the twenty-first century, diabetes mellitus has become an epidemic and being a risk factor of cardiovascular diseases [1] leads to an increased mortality in the population of developed and developing countries. Its severe complication—diabetic foot syndrome—is the primary cause of limb amputation due to vascular complications. An impaired wound healing is crucial in the clinical manifestation of diabetes, and hyperglycemia in uncontrolled diabetes is the major pathogenic factor.

The process of wound repair involves several consecutive phases (inflammatory and proliferative phase, remodeling of the wound), and it demands various factors: polypeptide growth factors, cytokines, and extracellular matrix components [2, 3]. Many of them are derived from endothelial cells, namely vascular endothelial growth factor (VEGF), tissue plasminogen activator (t-PA), metalloproteinases (MMP-2, MMP-9) and their inhibitors, tumor necrosis factor alpha (TNF-α), and interleukin-6 (IL-6). Therefore, the endothelium is essential in the maintenance of vascular homeostasis [4]. Hyperglycemia in diabetes is responsible for damaging of the endothelium and increases inflammation on the surface of the vascular lining [5].

TNF-α enhances the inflammation process and has an impact on angiogenesis and destructive processes. It induces the production of other pro-inflammatory cytokines, e.g., IL-6. It contributes to oxidative stress by generating reactive oxygen species (ROS) [6]. TNF-α is the most important pro-inflammatory factor in diabetes.

IL-6 is a pleiotropic cytokine with a wide spectrum of biological activity. Its main function is to participate in the immune response and acute phase of inflammatory reaction where it acts together with TNF-α [7]. In the acute inflammatory response, Il-6 has regulatory properties and limits the production of pro-inflammatory cytokines such as TNF-α [8]. IL-6 can therefore suppress the development of diabetes. However, when produced in excess and in long term, it promotes a passage of inflammation into chronic phase and contributes to the development of many diseases [9].

Many studies have shown that high glucose concentration (20–40 mM/L) in the culture medium imitates conditions as in uncontrolled diabetes and adversely affects cell proliferation leading to cell damage and apoptosis [10–12].

The number of patients suffering from diabetes is still increasing, and chronic complications of microangiopathy and macroangiopathy cause disability and inability to work and a deterioration of the quality of life. Diabetes is also the leading cause of amputation [13] due to non-healing wounds. The method of phototherapy using laser beam of low power (LLLT—low-level laser therapy) effectively supports conventional treatment and brings a significant improvement in the quality of life in diabetes.

According to Brosseau et al. [14], lasers have been used in medicine since at least 1974 when LLLT was officially implemented in the USSR as an alternative non-invasive treatment of rheumatoid arthritis. A number of studies have reported the positive impact of LLLT on alleviation symptoms caused by hyperglycemic conditions. These investigations involved humans and animals and were conducted in vitro [15–17] and in vivo [18–20]. Mainly, they evaluated fibroblasts and osteoblasts. Research from the last decade provides data on the key role of the vascular endothelium in maintaining vascular homeostasis and pathogenesis of vascular diseases. Endothelial cells line the walls of blood vessels and therefore are on the front line of contact with the high concentration of glucose.

The aim of our study was to evaluate the effect of low-power laser irradiation at two wavelengths (635 and 830 nm) on the secretion of inflammatory factors (TNF-α and IL-6) by the endothelial cell culture—HUVEC line (human umbilical vein endothelial cells)—under conditions of hyperglycemia.

## Material and methods

Endothelial cells (HUVEC line) were derived from human umbilical veins by the enzyme method using collagenase according to the method described by Jaffe et al. [21]. Cells were cultured in M199 media supplemented with 20 % fetal bovine serum (FBS), 100 U/ml penicillin (Gibco® products), and growth factors 50 μg/ml endothelial cell growth supplement (ECGS—Corning Inc. USA) and heparin. ECGS was obtained from a bovine neural tissue. The cells were incubated at 37 °C in a humidified atmosphere with 5 % CO_2_. After 2–4 passages and seeding the cells in 6-well culture plates, the proper experiment was conducted. HUVECs were placed at a density of 7.5 × 10^4^ cells per square centimeter.

With the exception of the control group, 30 mM/L glucose was added to the culture medium. The culture medium was changed every 3 days. The experiment was repeated three times with three independent cells isolations.

A semiconductor-based laser (Roithner Lasertechnik GmbH, Austria) was used to generate a visible laser beam with the wavelength of 635 nm (AlGaAlP) and the wavelength of 830 nm (GaAlAs) in the infrared. The authors have used the optoelectronic set for controlled, reproducible exposure of electromagnetic irradiation of biological structures in the spectral band of tissue transmission window 600–1000 nm [22]. The power of laser sources was 30 mW for 635 nm and 60 mW for 830 nm. The power density at the cell-layer level measured by using a laser power meter (Gentec, Model SOLO2 R2, Canada) was 1.875 mW/cm^2^ for 635 nm and 3.75 mW/cm^2^ for 830 nm. The power was constant in all experiments. The distance between the laser source and the surface of application was 10 cm, the application was carried through an optical fiber, and there was 80 cm^2^ of irradiated area. The experiment was conducted in four groups: 1—no glucose in culture medium, no irradiation (control group); 2—glucose, no irradiation; 3—glucose, laser irradiation with wavelength of 635 nm, energy dose of 2 J/cm^2^; and 4—glucose, laser irradiation with wavelength of 830 nm, energy dose of 2 J/cm^2^. The time of laser irradiation was 1066 s for 635 nm and 533 s for 830 nm. The cells were cultured for 7 days, with two irradiations on days 5 and 6. At the end of experiment, conditioned medium from each well of culture plates was collected and centrifuged for 10 min at 2000×*g* and frozen at −86 °C. After thawing, the concentration of TNF-α and IL-6 in the supernatant was measured by ELISA test (eBioscience, Vienna, Austria) according to the manufacturer’s instructions. The remaining cells on the bottom of each well were harvested by using trypsin and counted by Buerker hemocytometry. This method uses trypan blue dye according to the method described by Basso et al. [23]. The results of the concentration of the parameters in the supernatant from each well of culture plates were analyzed per number of cells in each well.

Statistical analysis was performed using Statistica 10.0 (StatSoft Inc.). The one-way ANOVA was used for parametrical analysis (proliferation), and Kruskal-Wallis test was used for nonparametrical comparisons (TNF-α and IL-6). Statistical significance was defined as *P* < 0.05. The results were presented as mean (M) ± standard deviation (SD) or median (Me), lower (Q_1_) and upper (Q_3_) quartile.

The approval of the Bioethics Commission of the NCU Collegium Medicum in Bydgoszcz was obtained, No KB/135/2009, date: 27 May 2014.

## Results

**Fig. 1:**
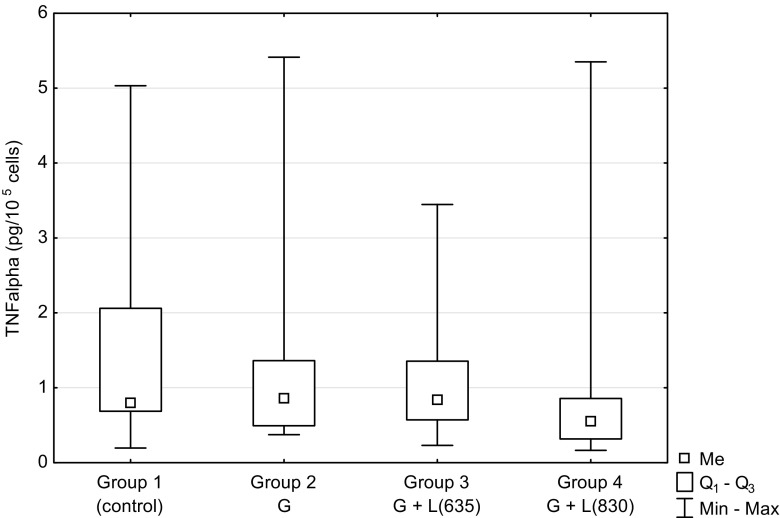
The concentration of tumor necrosis factor-alpha (*TNF-α*) in the supernatant of HUVEC cells culture depending on hyperglycemia and laser irradiation of different wavelengths. *G* glucose in culture medium, *L(635)* laser irradiation with wavelength of 635 nm, *L(830)* laser irradiation with wavelength of 830 nm. *P* value of ANOVA was 0.1033

Figure 1 shows the results concerning the effect of low-level laser therapy on TNF-α concentration in the supernatant from HUVEC culture which was grown in the high concentration of glucose in the culture medium (glucose concentration of 30 mM/L). The level of TNF-α in the control group (group 1) without glucose in the medium and without laser irradiation was 0.79 pg/10^5^ cells. Its concentration was only slightly higher (0.85 pg/10^5^ cells) in the unirradiated group 2 containing glucose in the medium. The cells from groups 3 and 4 were grown in medium with glucose and irradiated at the wavelength of 635 and 830 nm, respectively. The TNF-α level in group 3 was slightly lower and in group 4 notably lower when compared to the group 2 result. The concentration in group 4 was 0.55 pg/10^5^ cells and was about 35 % lower than in group 2. However, the final outcome of the ANOVA testing was statistically non-significant and reported as 0.1033.

**Fig. 2:**
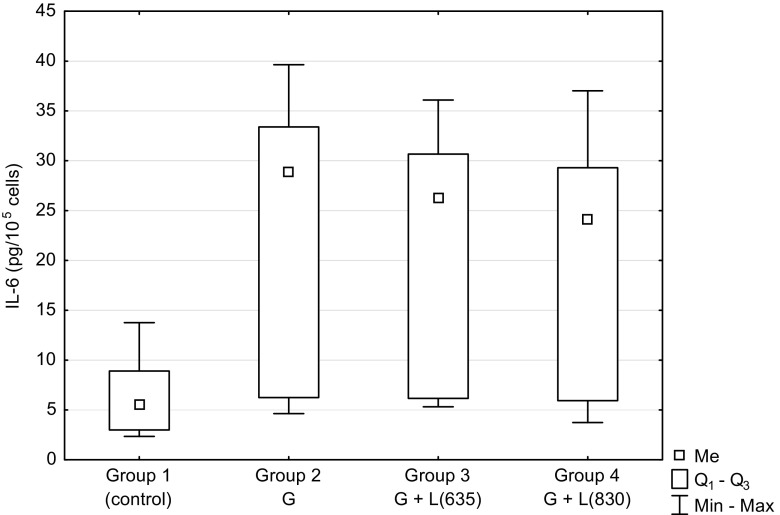
The concentration of Interleukin-6 (*IL-6*) in the supernatant of HUVEC cells culture depending on hyperglycemia and laser irradiation of different wavelengths. *G* glucose in culture medium, *L(635)* laser irradiation with wavelength of 635 nm, *L(830)* laser irradiation with wavelength of 830 nm. There was a statistically significant difference between the three experimental groups (2, 3, 4) and the control group (*P* = 0.0003, *P* = 0.0013, *P* = 0.0219, respectively)

Figure 2 presents the concentration of interleukin 6 in the corresponding groups as in Fig. 1. The concentration of IL-6 increased several times after the addition of glucose to the culture medium. There was a statistically significant difference between the three experimental groups (2, 3, 4) and the control group (*P* = 0.0003, *P* = 0.0013, *P* = 0.0219, respectively). No effect of the laser was observed. There was no increase of IL-6 concentration in groups 3 and 4 when compared to group 2. Difference between groups 2, 3, and 4 was statistically non-significant.

**Fig. 3:**
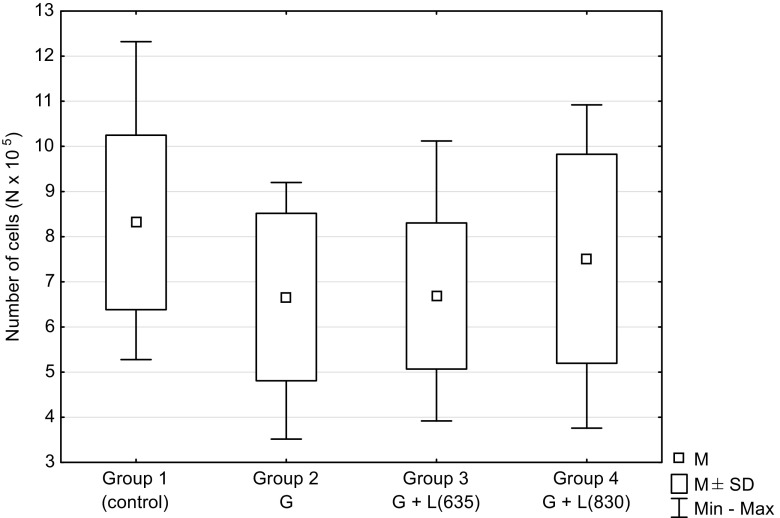
The number of HUVEC cells depending on hyperglycemia and laser irradiation of different wavelengths. G glucose in culture medium, *L(635)* laser irradiation with wavelength of 635 nm, *L(830)* laser irradiation with wavelength of 830 nm. There was a statistically significant difference between the two experimental groups (2, 3) and the control group (*P* = 0.0207, *P* = 0.0190, respectively)

Figure 3 shows the number of HUVECs which was the highest in the control group, while the lowest number was observed in group 2. This difference in relation to the control group was statistically significant (*P* = 0.0207). The number of cells in group 3 was slightly higher compared to group 2 and in group 4 reached the level similar to the control group.

## Discussion

High concentration of glucose present in diabetes causes damage to endothelial cells. It is accompanied by inflammation associated with the secretion of pro-inflammatory cytokines such as TNF-α and IL-6. An increased secretion of TNF-α and IL-6 under conditions of hyperglycemia is a finding observed by several authors [24–27]. It is accompanied by a reduction of cell proliferation [10, 11, 15]. However, in the study of Brandner et al. [28], level of TNF-α in cultured diabetic keratinocytes did not differ significantly from nondiabetic keratinocytes, although the level of TNF-α messenger RNA (mRNA) in the skin of patients with diabetes was significantly higher compared to those without diabetes. The authors explain this finding by the fact that keratinocytes are not a major producer of TNF-α in the skin. In our study, TNF-α was only slightly higher in cell cultures with the high glucose level in the medium compared to cells grown under normal conditions (Fig. 1). In contrast, the IL-6 level was significantly increased in the groups with glucose in the medium compared to the control group without glucose (*P* = 0.0003; Fig. 2). IL-6 can inhibit the production of TNF-α [8, 29] which is why a considerable increase of IL-6 under hyperglycemic conditions might have an impact on the level of TNF-α. Other authors have also observed the significant increase of IL-6 concentration under conditions of elevated glucose level [24, 30, 31]. In our study, a much smaller number of endothelial cells cultured under hyperglycemic conditions compared to culture under normal conditions (*P* = 0.0207, Fig. 3) shows the negative impact of high glucose concentration on the proliferation and cell viability, as reported also by other authors [10, 12].

In hyperglycemia, glucose is subject to auto-oxidation process and non-enzymatic glycation which leads to the production of reactive oxygen species. An increased oxidation of the cofactor NADPH to NADP+ and the reduction of NAD+ to NADH are observed [32]. These reactions disrupt a balance between oxidants and antioxidants system leading to hypoxia and synthesis of advanced glication end-product (AGE). It is the effect of cytokines, mainly TNF-α [26]. Hyperglycemia increases the production of free radicals in the mitochondria, particularly superoxide anion. These processes exacerbate the oxidative stress [33–35] which damages cells and induces apoptosis [36, 37]. Apoptosis may be promoted also by DNA damage [11, 38] and irreversible changes in cytoskeletal organization at high glucose concentration [39, 40]. IL-6 secreted during the acute phase of inflammation has varied effects on cells in diabetes [30]. Pro-inflammatory or anti-inflammatory properties of IL-6 depend on the nature and function of cells and factors inducing inflammation. At high concentration, it can inhibit the production of TNF-α [41] which was confirmed in studies in mice [42]. The cited works have shown that the reduced level of IL-6 is associated with the impaired wound healing in diabetes.

Hyperglycemia induces the production of pro-inflammatory cytokines and growth factors by activating key signaling pathways associated with MAPK (mitogen-activated protein kinases), NF-κB (nuclear factor-κB), and STAT3 (signal transducers and activators of transcription) [27, 31, 43, 44] depending on ROS and oxidative stress. This is related to the effect of TNF-α on altering the redox reaction in the mitochondrial respiratory chain [35, 45, 46].

Although molecular mechanisms of low-power laser irradiation are still poorly understood, it is already known that the beneficial effects are associated with stimulation of cellular metabolism after energy absorption [47]. A stimulation of photoreactive proteins like cytochrome C occurs in the mitochondrial respiratory chain. This can increase the availability of ATP in the cells and have an influence on reactive oxygen species [48, 49]. An improvement of the cellular energy state and normalization of cellular functions is manifested by alleviating the pain and inducing the self-healing process [50]. Kwon [16] has found that laser irradiation of osteoblasts in a diabetic model at the wavelength of 635 nm reduces the level of generated ROS.

Studies in animals suggest beneficial effects of LLLT in case of hyperglycemia. LLLT (wavelength of 650 and 980 nm) in rats with induced diabetes has an antihyperglycemic effect without an impact on the blood morphology and biochemical profile [19]. Rabelo et al. [18] have also reported the beneficial effect of reduced inflammation in diabetic rats irradiated by HeNe laser at 632.8 nm.

The effects of LLLT on various types of cells depend on irradiation parameters such as dose and wavelength. Higher doses (10 or 16 J/cm^2^) cause reduction of cell viability and mitochondrial activity and increase the percentage of DNA damage [50, 51]. The wavelength is also important during irradiation of diabetic fibroblast cultures. The wavelength of 1064 nm gave significantly worse results in wound healing than 632.8 and 830.0 nm [51, 52].

Our research confirms the beneficial effects of LLLT on cells cultured with high concentrations of glucose. TNF-α level in the group of cells cultured in medium containing high concentration of glucose decreased under the influence of laser irradiation when compared to the unirradiated group. This applies particularly to 830 nm (Fig. 1). *P* = 0.1030 is not far from significance. This result of testing in the analysis of variance could be significantly influenced by values in group 4 (glucose in the medium, LLLT *λ* = 830 nm) with an average value of TNF-α lower about 35 % in comparison to group 2 (glucose in the medium, without irradiation). Many authors have also observed TNF-α reduction caused by laser irradiation [53, 54].

Our results show the significant increase of IL-6 induced by hyperglycemia (*P* = 0.0002); LLLT did not cause significant changes in the concentration of this cytokine in the endothelial cell culture (Fig. 2). Some authors have reported a rise in the level of IL-6 induced by LLLT [30, 55, 56]. Houreld et al. [53] have observed only slight and statistically non-significant increase of IL-6. Some of the literature data have also reported decreased level of IL-6 under the influence of laser irradiation [56]. The result we have obtained may be in favor of beneficial effects of LLLT, since IL-6 reducing TNF-α level can therefore protect cells from the damaging effect of hyperglycemia. This issue is discussed and needs further research [8, 57].

Data from the literature suggest that LLLT promotes the metabolic activity of cells and stimulates their proliferation [15, 58], in case of cells grown under hyperglycemic condition as well [50, 52, 53]. These findings are consistent with our research applying in particular 830 nm (*P* = 0.0190; Fig. 3). Hyperglycemic conditions significantly reduce the proliferation of HUVECs. The use of laser irradiation increases the proliferation of these cells to the level observed in the control group (non-irradiated cells in medium without glucose) (Fig. 3).

## Conclusion

1. It appears that the adverse effects of hyperglycemia on vascular endothelial cells may be corrected by treatment with LLLT, especially at the wavelength of 830 nm. It causes the reduction of TNF-α concentration and enhancement of cell proliferation.
2. Due to the significance of endothelial cells in the pathogenesis of diabetes and a small amount of studies evaluating the impact of LLLT on these cells under conditions of hyperglycemia, further work on this subject is warranted.
